# Copper-mediated chemodynamic therapy with ultra-low copper consumption by doping cupric ion on cross-linked (*R*)-(+)-lipoic acid nanoparticles

**DOI:** 10.1093/rb/rbad021

**Published:** 2023-03-23

**Authors:** Rong Cui, Bing Li, Chunyan Liao, Shiyong Zhang

**Affiliations:** College of Biomedical Engineering, National Engineering Research Center for Biomaterials, Sichuan University, Chengdu 610064, China; Hubei Key Laboratory of Wudang Local Chinese Medicine Research, School of Pharmaceutical Sciences, Hubei University of Medicine, Shiyan 442000, Hubei, China; College of Biomedical Engineering, National Engineering Research Center for Biomaterials, Sichuan University, Chengdu 610064, China; College of Biomedical Engineering, National Engineering Research Center for Biomaterials, Sichuan University, Chengdu 610064, China

**Keywords:** chemodynamic therapy, copper metabolism, (R)-(+)-lipoic acid, nanodrug

## Abstract

Cu-mediated chemodynamic therapy (CDT) has attracted prominent attention owing to its advantages of pH independence and high efficiency comparing to Fe-mediated CDT, while the application of Cu-based CDT agents was impeded due to the high copper consumption caused by the metabolism loss of copper and the resultant potential toxicity. Herein, we developed a new copper-mediated CDT agent with extremely low Cu usage by anchoring copper on cross-linked lipoic acid nanoparticles (Cu@cLAs). After endocytosis into tumor cells, the Cu@cLAs were dissociated into LA and dihydrolipoic acid (DHLA) (reduced form of LA) and released Cu^2+^ and Cu^+^ (oxidized form of Cu^2+^), the two redox couples recycled each other in cells to achieve the efficient killing of cancer cells by delaying metabolic loss and increasing the ROS level of tumor cells. The self-recycling was confirmed in cells by the sustained high Cu/DHLA content and persistent ROS generation process. The antitumor study based on the MCF-7/R nude mice gave the Cu@cLAs a tumor inhibitory rate up to 77.9% at the copper of 0.05 mg kg^−1^, the first dosage reported so far lower than that of normal serum copper (0.83 ± 0.21 mg kg^−1^). This work provides not only a new promising clinical strategy for the copper excessive use in copper-mediated CDT, but also gives a clue for other metal mediated disease therapies with the high metal consumption.

## Graphical Abstract

**Figure:**
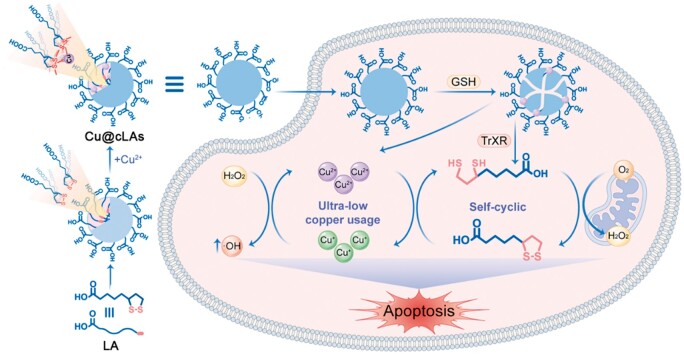

## Introduction

As an emerging therapeutic approach for cancer treatment, the chemodynamic therapy (CDT) has attracted prominent attention owing to its advantages of high selectivity and endogenous activation [1–3]. Currently, the conversion of endogenous hydrogen peroxide (H_2_O_2_) into highly toxic hydroxyl radical (•OH) by iron-mediated Fenton reaction is the main strategy to achieve CDT [4, 5]. However, in general, Fe^2+^-catalyzed Fenton reaction agents have low efficiency at eradicating cancer cells due to their need to operate in highly acidic environments (pH 2–4) [6, 7]. In this case, some acid-independent Fenton agents, such as Cu, have been gradually developed. The Cu^+^-catalyzed Fenton-like reactions are efficient in both slightly acidic and neutral environments with high reaction rates, and thus have great potential as CDT catalysts for cancer treatment [8, 9]. Currently, various Cu-containing CDT agents, such as CuO_2_ [10], bimetallic Cu^2+^ complex [11], copper-cysteine mercaptide nanoparticles [12], have been reported and demonstrated the good antitumor effect on some cancers. Unfortunately, due to persistent oxidative stress and the resultant high metal metabolic loss, these good anticancer effects were achieved at the expense of high copper consumption which might cause the risk of a variety of diseases, such as acute Cu poisoning, Alzheimer’s disease and vascular diseases [13–15]. Even the reported highly efficient amorphous copper nanoparticle system requires 1.6 mg copper of per kg mice to achieve satisfying therapeutic efficacy [12], significantly higher than that of normal mice’s serum copper [16].

Lipoic acid (LA), a member of the B vitamin group, has been widely used in nutraceuticals for its 400-fold higher antioxidant capacity than vitamin C. In addition, LA and its reduced form, dihydrolipoic acid (DHLA), have been found to induce apoptosis in various cancer cells without affecting nontransformed normal cells [17]. From the perspective of molecular structure, LA is an amphiphilic small molecule compound constructed by a hydrophobic disulfide five-membered ring and a hydrophilic carboxyl group, which can be easily assembled into nanoparticles in water [18–20]. Meanwhile, the reduction responsiveness of the disulfide bond and the pH sensitivity of the carboxyl group can bring unique advantages to the application of nanoparticles in drug delivery [21]. Based on the above structural characteristics and functional advantages, our group has developed various cross-linked LA nano-carriers constructed only by LA [17, 22–24]. Benefiting from the structural homology with LA (both are converted to DHLA in cells), these nano-carriers not only have good biosafety, but also exhibit antitumor activity similar to LA. These carriers with synergistic antitumor effect represent a promising platform for drug delivery [17].

**Scheme 1.**
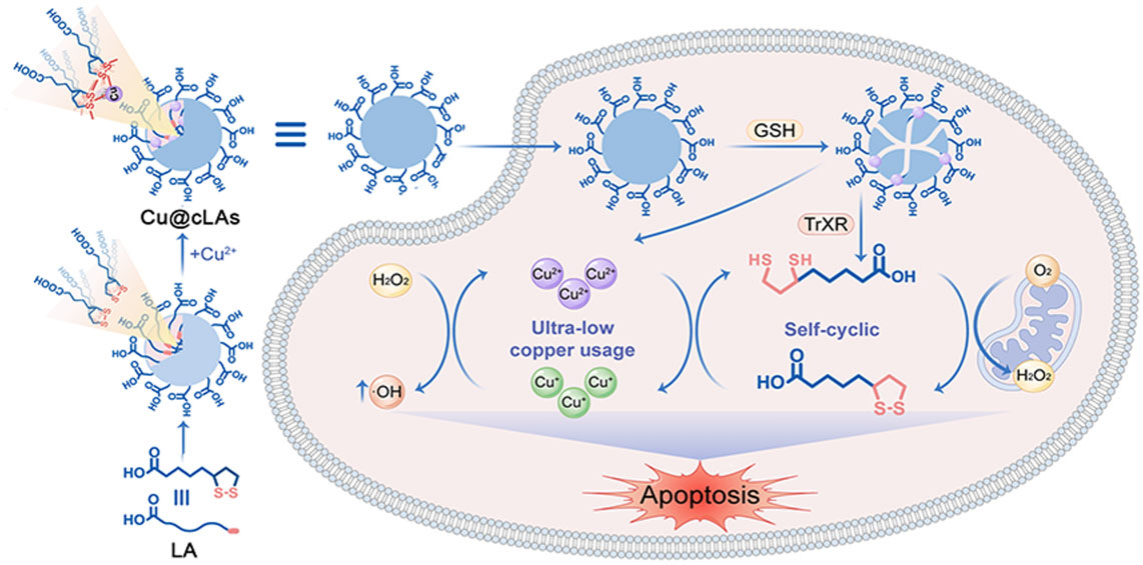
Schematic illustration of Cu@cLAs based self-cyclic system for copper-mediated CDT.

Herein, we develop a new copper-mediated CDT agent with extremely low Cu usage by anchoring copper on cross-linked LA nanoparticles. According to Scheme 1, after entering tumor cells, the Cu@cLAs degrades to DHLA and releases Cu^2+^. Since LA/DHLA holds a low redox potential, DHLA can reduce copper ions from high valence to low valence, thus providing a continuous supply of catalyst for Fenton-like reaction. Meanwhile, the conversion between Cu^2+^/Cu^+^ enhances the closed loop of LA and DHLA, which would delay their metabolic loss and increase the ROS level in tumor cells. The *in vitro* results showed that the Cu@cLAs could induce a persistent ROS generation process. The antitumor experiment based on the MCF-7/R nude mice model showed that the Cu@cLAs inhibited tumors up to 77.9% at the dose of 5 mg kg^−1^, which was much higher than that of DOX (5 mg kg^−1^, 42.9%). Significantly, the copper content contained in the dose to achieve such high curative effect is as low as 0.05 mg kg^−1^, far lower than that of normal serum copper (0.83 ± 0.21 mg kg^−1^). This self-cyclic strategy of Cu thus delicately overcomes the problem of high metabolic loss of Cu, thus showing the great potential in clinic.

## Experimental

### Synthesis of Cu@cLAs

(R)-(+)-LA (50 mg, 0.24 mmol) solution of acetone (5 ml) was dropped into 50 ml of room temperature oscillating deionized water. In the following step, acetone was evaporated using a rotary evaporator. The resulting solution was then irradiated with 365 nm UV light for ∼4 h, and an aqueous solution of CuCl_2_·2H_2_O (50 µl, 1 mol l^−1^) was immediately added gently under stirring, followed by the ring-opening polymerization under UV irradiation. Ultimately, the resulting mixture was vigorously blown through an air-blowing device, and then dialyzed in distilled water for 48 h to extract the Cu@cLAs.

### In vitro degradation assay

The Cu@cLAs degradation was assumed to start as soon as the dialysis bags were placed into the reservoir. Briefly, 2.0 ml of Cu@cLAs were infused into 48 ml PBS (pH = 7.4) with 10 mM GSH in dialysis bags (1.0 kDa MWCO). Periodically, 0.5 ml of the solution was taken from the reservoir, and the amounts of released Cu@cLAs were analyzed by dynamic light scattering (DLS) with count rate.

### Detection of intracellular ROS concentration changes

MCF-7/R cells were planted and incubated with Cu^2+^, cLAs and Cu@cLAs for different times in the 24-well plate, respectively, and the times were set as 0, 1, 2, 4 and 6 h. After incubation, the cells were stained by adding the DCFH-DA fluorescent probe, and the fluorescence intensity was detected by a fluorescence microplate reader, and a graph of the change in fluorescence intensity over time was drawn. Taking Cu@cLAs group as an example, the ROS intensity of 0 h was set as 100%, and the relative ROS level of Cu@cLAs after incubation for 12 h was calculated by the following equation:


$$\mathrm{Relative}\;\mathrm{ROS}\;\mathrm{level}=\;\frac{\left[\mathrm{ROS}\;\mathrm{level}\;\mathrm{of}\;\mathrm{Cu}@\mathrm{cLAs}/12\;h\right]}{\left[\mathrm{ROS}\;\mathrm{level}\;\mathrm{of}\;\mathrm{Cu}@\mathrm{cLAs}/0\;h\right]}\times \;100\%.$$

### Intracellular detection of Cu content

An incubation period of 12 h was conducted on MCF-7/R cells seeded in six-well plates at a density of 10^6^ cells. Then the medium was replaced by a fresh one containing 10 μM Cu@cLAs, Cu^2+^ and the cells were incubated for 0, 0.5, 1, 2, 3, 4, 5 and 6 h at 37°C in an incubator with 5% CO_2_. After discarding the medium, the cells were washed three times with PBS before being harvested. Nitric acid, 30% H_2_O_2_ and concentrated hydrochloric acid were used to digest the cells. The content of copper in the whole cells was determined by ICP-MS.

### Intracellular detection of DHLA content

An incubation period of 12 h was conducted on MCF-7/R cells seeded in six-well plates at a density of 10^6^ cells. Then the medium was replaced by a fresh one containing 10 µM Cu@cLAs, cLAs and the cells were incubated for 0, 1, 2, 4, 6 and 12 h at 37°C in an incubator with 5% CO_2_. After discarding the medium, the cells were washed three times with PBS before being harvested. The content of DHLA in the whole cells was determined by HPLC–MS.

### Biodistribution study

Biodistribution studies were performed by tail vein injection of Cu@cLAs into MCF-7/R tumor-bearing mice. Mice were sacrificed after 24 h post-injection, the tumors and major organs were collected, weighed and digested in nitric acid, followed by measurement of copper content by ICP-OES. Mice injected with saline served as a control.

## Results and discussion

### Synthesis and characterization of Cu@cLAs

**Figure 1.**
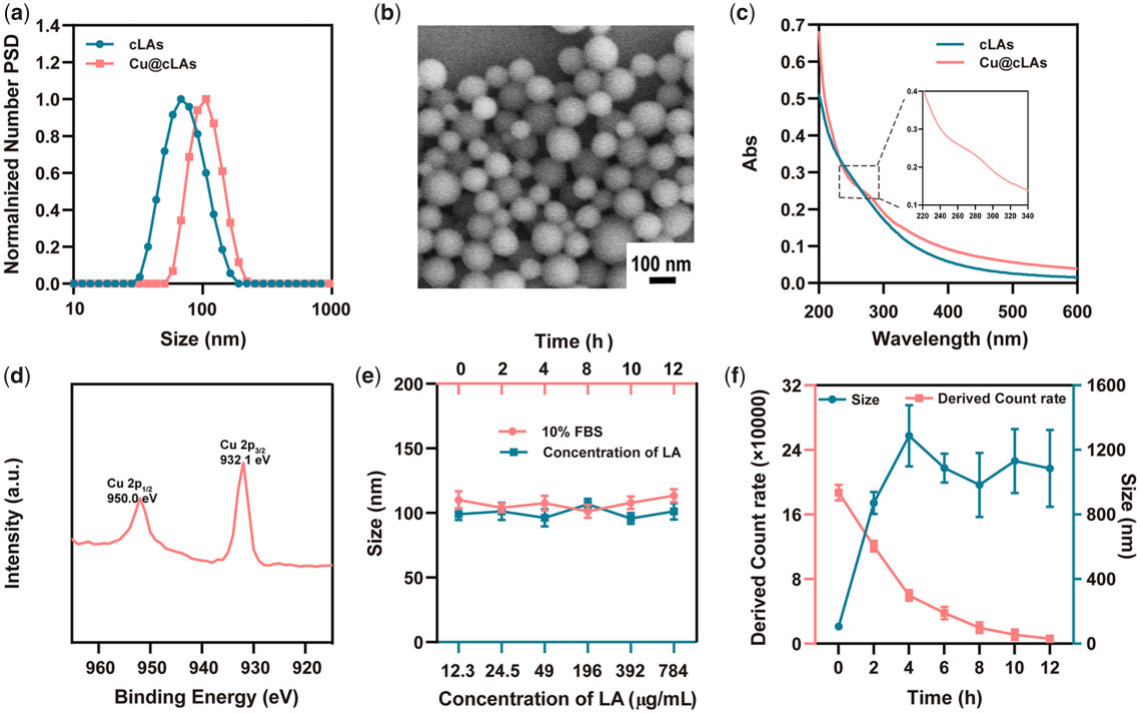
Synthesis and characterization of Cu@cLAs. (**a**) Distribution of the hydrodynamic diameter of cLAs, Cu@cLAs (S/Cu = 100:1, wt/wt) measured by DLS. (**b**) SEM micrographs of Cu@cLAs. (**c**) UV–vis spectra of cLAs and Cu@cLAs. The inset shows the regional enlarged spectra of Cu@cLAs. (**d**) XPS spectrum of the Cu@cLAs. (**e**) Particle size of Cu@cLAs incubated with 10% FBS as a function of time and size of Cu@cLAs as a function of the concentration of LA. (**f**) Particle size and derived count rate of Cu@cLAs incubated with 10 mM GSH as function of time.

The cLAs was prepared simply by dropwise adding acetone-dissolved LA into water, followed by irradiation under 365 nm UV light and dialysis against deionized water. The Cu@cLAs were synthesized by reacting CuCl_2_ with sulfhydryl group formed by LA upon UV light, where a color change from a pale blue to light yellow occurred after adding copper. The successful encapsulation of Cu was characterized by energy dispersive spectroscopy (Supplementary Fig. S1), and the loading amount was determined via inductively coupled plasma-atomic emission spectroscopy (ICP-AES). The DLS measurements showed that the Cu@cLAs exhibited a marginal increase in size from before copper-adding ∼78.1 nm to after copper-adding ∼106 nm and zeta potential from before copper-adding −22.3 mV to after copper-adding −17.6 mV (Fig. 1a; Supplementary Fig. S2 and Table S1). The Cu@cLAs with comparable diameters to the DLS demonstrated a spherical morphology under the scanning electron microscopy (SEM) characterization (Fig. 1b). To confirm that copper ions was a coordinated with sulfhydryl group-containing ligands, the UV absorption was performed, which showed that a new absorption peak appears at around 280 nm after copper addition, indicating a characteristic belonging to the Cu–S bond (Fig. 1c) [25, 26]. In the Cu@cLAs solution, the low content of Cu and the presence of nanoparticles could explain this inconspicuous shoulder peak at 280 nm. Further confirmation of the Cu–S bond was confirmed by X-ray photoelectron spectroscopy (XPS), with peaks at 570 eV attributed to Cu^+^ and at 952 and 932.1 eV to Cu^2+^ (Fig. 1d).

Next, the stability of the NPs was assessed. The particle size remains unchanged when Cu@cLAs concentration is below critical aggregation concentration or when co-incubated with 10% fetal bovine serum (FBS) solution (Fig. 1e) [24]. Importantly, the Cu@cLAs held a constant size in PBS and DMEM (Supplementary Fig. S3), suggesting the good stability of Cu@cLAs in complex physiological environment. When co-incubated with 10 mM glutathione (GSH) for 12 h, the size of nanoparticles grew to 1085 nm and its count rate decreased from 180 000 to 5000 (Fig. 1f; Supplementary Fig. S4), demonstrating the selective degradation properties under tumor redox conditions.

### In vitro anticancer evaluation of Cu@cLAs

We evaluated the *in vitro* antitumor effect of Cu@cLAs using methyl thiazolyl tetrazolium assay on 4T1 cells, B16 cells and MCF-7/R cells, and the corresponding half inhibitory concentrations (IC_50_) are summarized (Table 1; Supplementary Table S2). Forty-eight-hour dose-dependent cell inhibition assays were conducted. The Cu^2+^ exerted high cytotoxicity to MCF-7/R tumor cells with an IC_50_ of 183.16 μM. To our delight, when cLAs is loaded with copper, the antitumor potency was greatly improved. Concretely, the Cu@cLAs achieved the ${\mathrm{IC}}_{50}^{\mathrm{Cu}}$ as low as 1.32 μM, which are up to 138 times lower than that of free Cu^2+^ (Supplementary Fig. S5). Notably, Cu@cLAs showed superior efficacy in killing tumor cells to other Cu-containing counterparts. For example, the ${\mathrm{IC}}_{50}^{\mathrm{Cu}}$ of Cu@cLAs on MCF-7/R cells is 1.32 μM, obviously lower than that of ${\mathrm{IC}}_{50}^{\mathrm{Cu}}$ of Cu-Cys NPs on MCF-7/R cells (250 μM), the optimal MCF-7/R cytotoxic copper-based CDT agent reported so far [12]. Meanwhile, it also the best cytotoxicity reported up now in using copper as the main CDT agent to treat tumors even extending toward all cell lines [27].

**Table 1.** IC_50_ of Cu^2+^, cLAs and Cu@cLAs with various Cu/LA ratios against MCF-7/R cells at 37°C for 48 h

Drug formulation	Cu/LA (mol mol^−1^)	${\mathrm{IC}}_{50}^{\mathrm{Cu}}$ (×10^−6^ M)
CuCl_2_	–	183.16
cLAs	–	–
Cu@cLAs	1:200	3.057
Cu@cLAs	1:150	2.106
**Cu@cLAs**	**1:100** ^a^	**1.32**
Cu@cLAs	1:80	3.684
Cu@cLAs	1:50	4.267

a The optimal synergy effect was obtained at the Cu/LA ratio of 1:100, where the labels were highlighted by bold format.

We further studied the impact of copper loading on the anticancer effect. One can found that as the load increased, ${\mathrm{IC}}_{50}^{\mathrm{Cu}}$ of Cu@cLAs shifted toward the trend of first decreasing and then increasing, suggesting that the ratio of Cu to cLAs has an important influence on the anticancer effect (Table 1). Consistent with the cytotoxicity results, the live/dead cell staining showed that only a small percentage of MCF-7R cells survived after treatment with Cu@cLAs for 24 h (Supplementary Fig. S6). A similar outcome was found in the western blot results, the band of Bcl-2 (the typical antiapoptotic protein) had nearly disappeared for the Cu@cLAs group (Supplementary Fig. S7). All in all, these results implied that the Cu@cLAs possessed higher lethality toward cancer cells.

### Anticancer mechanism of Cu@cLAs

**Figure 2.**
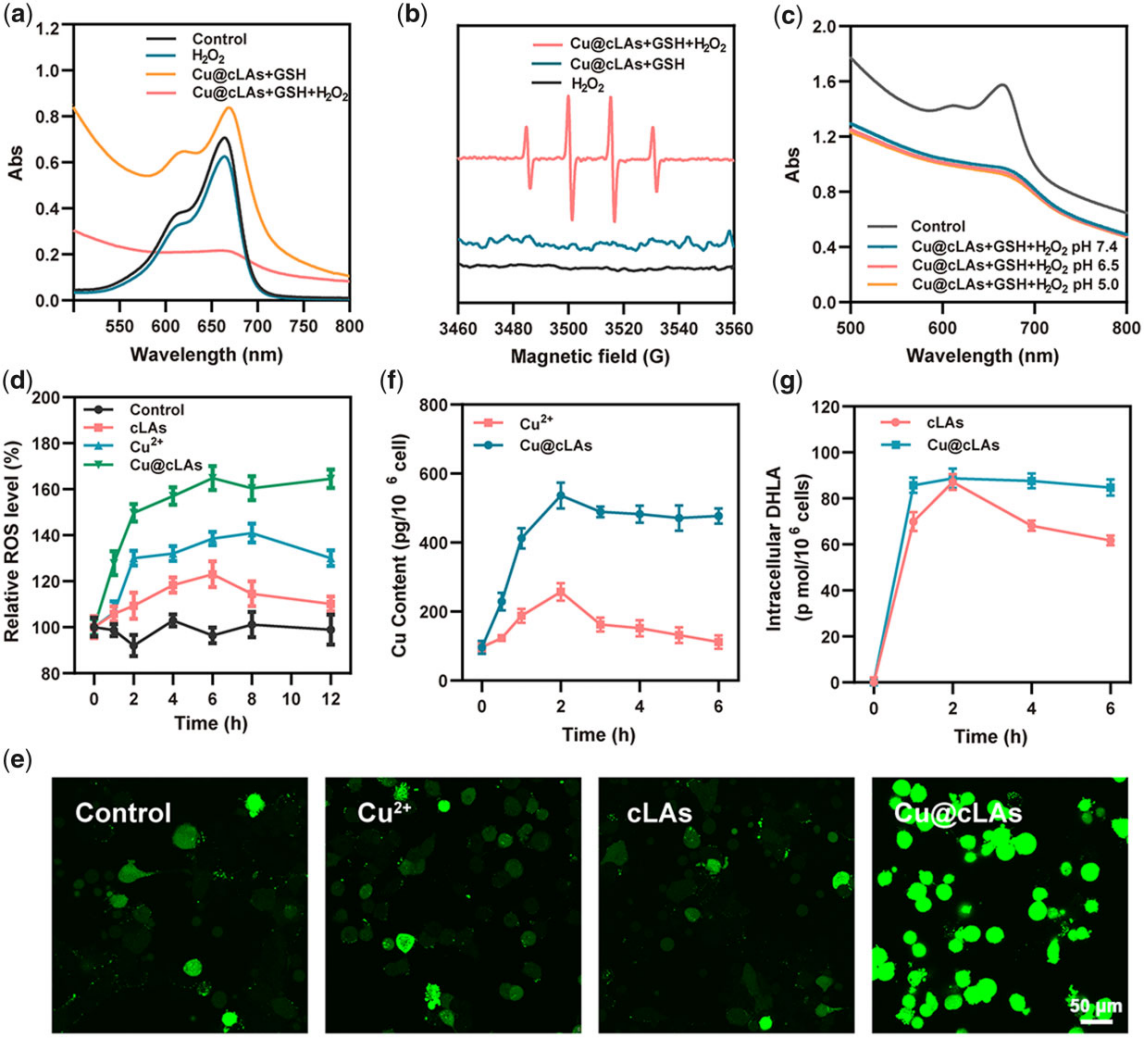
Anticancer mechanism of Cu@cLAs. (**a**) The degradation of MB by ROS under different conditions at pH = 7.4. The reaction time = 4 h. (**b**) ESR spectra of different groups after addition of DMPO. (**c**) MB degradation rate under different pH conditions. (**d**) Relative ROS level of MCF-7/R cells after incubation with different formulations at indicated time. (**e**) CLSM images of ROS production in MCF-7/R cells incubated with the indicated materials for 24 h. (**f**) Content change of Cu in MCF-7/R cells over time after incubation with Cu^2+^ and Cu@cLAs, respectively. (**g**) Content change of DHLA in MCF-7/R cells over time after incubation with cLAs and Cu@cLAs, respectively.

To verify that the effective antitumor effect is caused by the hypothesized self-cyclic mechanism (Scheme 1), the capability of Cu@cLAs in production of ROS in solution was explored firstly by using MB assay, since ROS can oxidize and degrade MB, decreasing its absorption intensity based on the fact that MB can be oxidized and degraded by ROS, resulting in a decreased absorption value [28, 29]. As shown in Fig. 2a, mere H_2_O_2_ or Cu@cLAs with GSH cannot degrade MB. In contrast, the absorption of MB decreased significantly after incubation of Cu@cLAs with GSH and H_2_O_2_. The degradation process of MB over time illustrated that MB had almost completely degraded after 90 min after incubation of Cu@cLAs with GSH and H_2_O_2_ (Supplementary Fig. S8), indicating that Cu@cLAs could efficiently generate ROS with the assistance of endogenous GSH and H_2_O_2_. To further determine the type of ROS, the electron spin resonance (ESR) spectroscopy was conducted. As shown in Fig. 2b, one can find that the characteristic 1:2:2:1 signals of •OH were only observed in Cu@cLAs with GSH and H_2_O_2_, indicating the efficient formation of •OH by Fenton-like reaction. Besides, we also checked the reaction rates at the different pH values (Fig. 2c). As a result, the different pH values did not affect the ROS production of Cu@cLAs. It is due to this property that Cu@cLAs-mediated CDT is highly efficient in producing ROS.

After confirmation of ROS production of the Cu@cLAs in the solution, the intracellular ROS production was monitored as a function of time. As observed in Fig. 2d, the treatment of MCF-7/R cells by Cu@cLAs resulted in higher level of ROS production than that of other groups, and the high ROS level could be maintained for more than 12 h while free cLAs and Cu^2+^ groups began to decline at 6 and 8 h, respectively. To our delight, even after incubation over 24 h, the Cu@cLAs-treated group exhibited still a strong green fluorescence, implying the continuous production of ROS, while the fluorescence signal in in the Cu- and cLAs-treated groups became weak (Fig. 2e; Supplementary Fig. S9). In addition, the intracellular H_2_O_2_ level in the Cu@cLAs group peaked at 6 h and then remained high until 12 h as opposed to the low and unchanged intracellular H_2_O_2_ level in the control group (Supplementary Fig. S10). These results implied the ability of Cu@cLAs in the persistent production of significant ROS.

To verify that the persistent-high ROS production was contributed to the self-cyclic regeneration of Cu and DHLA, their intracellular levels were monitored as a function of time. As plotted in Fig. 2f and g, when Cu@cLAs were incubated with MCF-7/R cells, the intracellular Cu and DHLA concentrations increased and peaked at 2 h. After that, they kept at high levels over a long time while the Cu and DHLA in other groups were largely metabolized, evidencing the continuous regeneration of Cu and DHLA in the group of Cu@cLAs.

### Biocompatibility of Cu@cLAs

**Figure 3.**
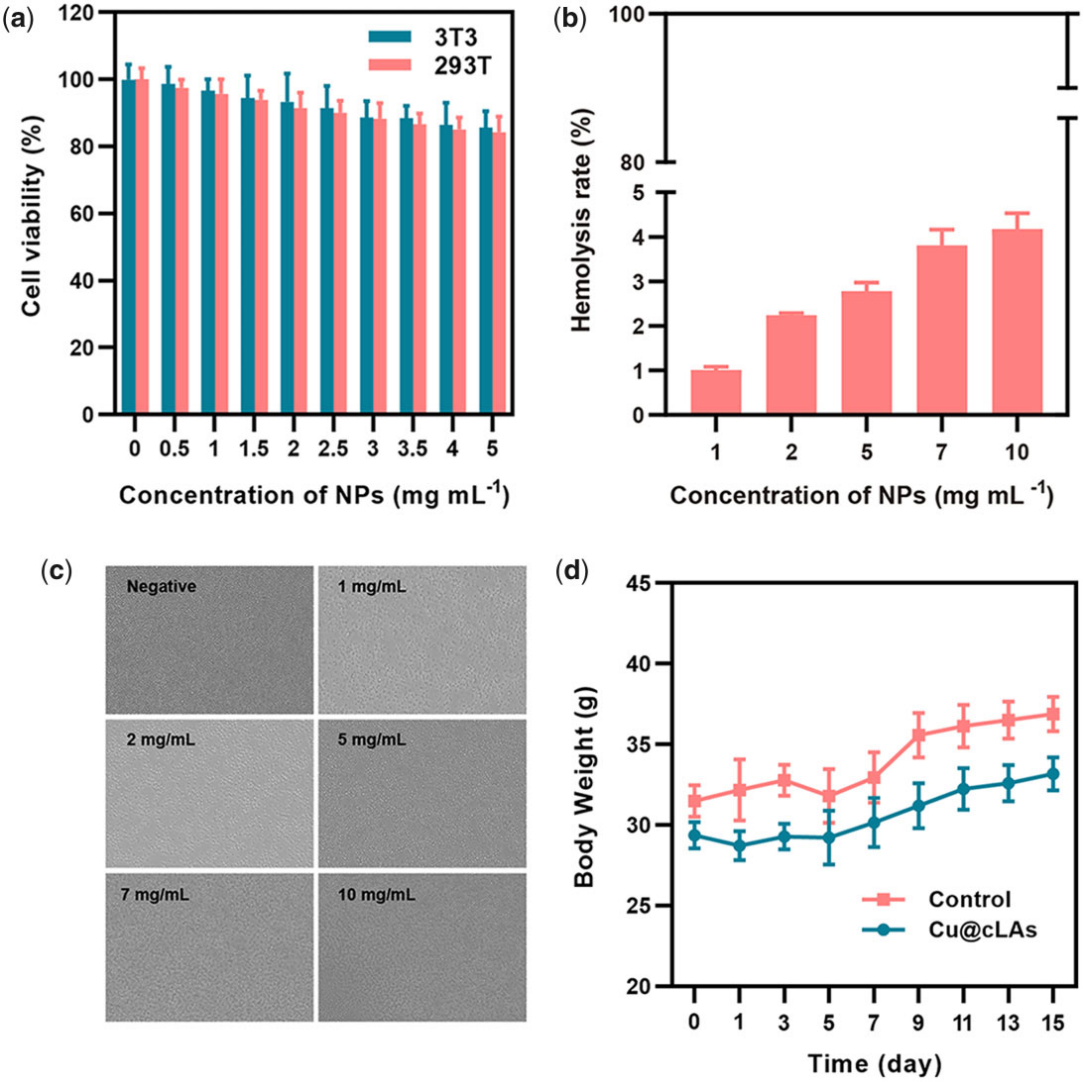
Biocompatibility of Cu@cLAs. (**a**) Cell viability of 293T and 3T3 cells after incubated with Cu@cLAs at various concentrations for 48 h (mean ± SD, *n* = 5). (**b**) Hemolysis rate of Cu@cLAs at different concentrations. Saline and distilled water were used as negative and positive controls, respectively. (**c**) Hemagglutination assay of Cu@cLAs at different concentrations. Saline was used as control. (**d**) Mouse weight change after intravenous injection administration of 50 mg kg^−1^ Cu@cLAs or saline (mean ± SD, *n* = 5). Saline was used as control.

We test the biocompatibility of Cu@cLAs both *in vitro* and *in vivo*. The *in vitro* biocompatibility was determined by measuring cytotoxicity and blood compatibility. According to Fig. 3a, the Cu@cLAs showed no cytotoxicity even at 5 mg ml^−1^ for mouse embryonic fibroblasts (3T3) and human renal epithelial cells (293T). Moreover, the Cu@cLAs gave almost no hemolysis (hemolysis ratio <5%; Fig. 3b) and cruor (Fig. 3c) even at the highest tested concentration (10 mg ml^−1^).

The *in vivo* biocompatibility of Cu@cLAs was evaluated by acute-toxicity assay. The acute toxicity was performed in BALB/c mice at a high dose of 50 mg kg^−1^ by observing adverse effects within a 14 days recovery time. During the 14 days, the mice did not show body weight loss or behavior disorders compared with the control group (Fig. 3d). The biochemistry analysis (Supplementary Table S3) and histological examination (Supplementary Fig. S11) revealed no acute toxic effects at tested concentration, indicating that Cu@cLAs possessed good biocompatibility.

### In vivo anticancer evaluation of Cu@cLAs

**Figure 4.**
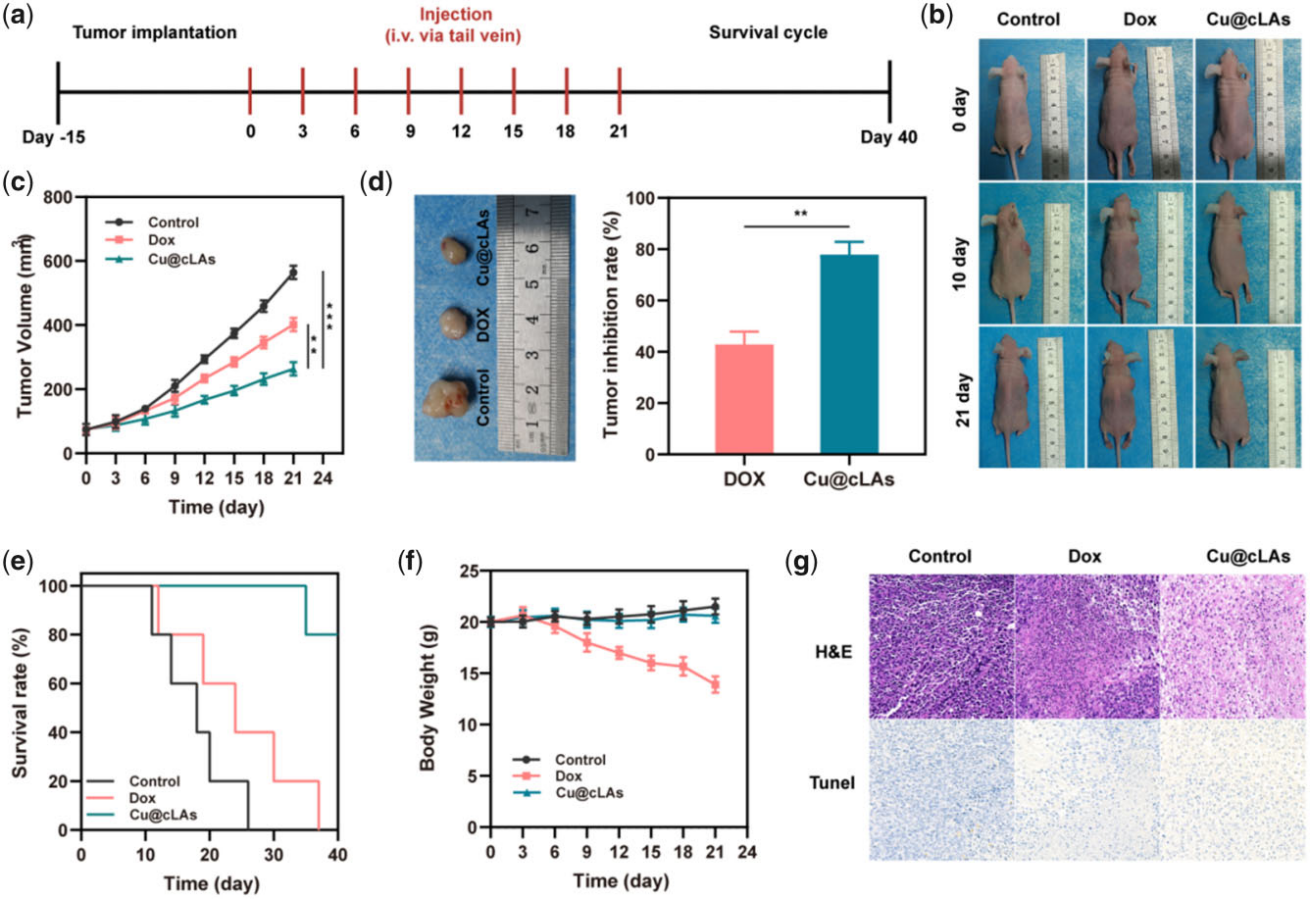
*In vivo* anticancer evaluation of Cu@cLAs. (**a**) Schematic illustration of MCF-7/R tumor xenograft construction, medication, and survival cycle. (**b**) Photos showing the tumor size in mice during different treatments. (**c**) Tumor volume change during therapy. (**d**) Digital pictures of tumors from the MCF-7/R tumor-bearing mice after 21 days of therapy and tumor inhibition rate of each therapeutic group after treatment. (**e**) Percent survival of mice after different treatments. (**f**) Body weight changes of the mice during therapy. (**g**) H&E staining and TUNEL staining of tumor sections from the MCF-/7R tumor-bearing mice.

Before *in vivo* anticancer evaluation, the *in vivo* biodistribution of Cu@cLAs in tumor-bearing mice was investigated based on the Cu content measured by inductively coupled plasma-optical emission spectrometry (ICP-OES). The Cu@cLAs with a small hydrodynamic can be effectively accumulated in tumor tissue though the EPR effect, as exhibited in Supplementary Fig. S12, Cu levels in tumor tissue were higher than those in liver (0.69 μg of Cu g^−1^ of tissue) and kidney (1.2 μg of Cu g^−1^ of tissue) when mice were treated with Cu@cLAs, implying that the nanoparticles would play a better role in subsequent antitumor applications. The animal model of MCF-7/R in the nude mice was employed to study the *in vivo* therapeutic efficacy of intravenously injected Cu@cLAs (5 mg kg^−1^, 10% of the safe dose (50 mg kg^−1^) of the Cu@cLAs) (Fig. 4a). As expected, during the 21-day therapeutic process, the saline and first-line chemotherapy drug DOX group (5 mg kg^−1^) showed a fast tumor growth rate in this tumor. In contrast, the tumor sizes in the Cu@cLAs group had only slightly increased from ∼50 to ∼200 mm^3^ (Fig. 4b and c). A further validation of Cu@cLAs superiority in tumor therapy was accomplished by excising, photographing and weighing all tumors following treatment (Fig. 4d). This result showed that the Cu@cLAs had a higher efficacy of tumor suppression (77.9% inhibition rate) than the chemotherapeutic drug DOX (42.9% inhibition rate). Importantly, the copper content of 5 mg kg^−1^ Cu@cLAs is as low as 0.05 mg kg^−1^, up to 32-fold lower than copper content of optimal counterparts (1.6 mg kg^−1^) reported so far. Encouragingly, the therapeutic efficacy was also verified by hematoxylin and eosin (H&E) and TdT-mediated dUTP-biotin nick end labeling (TUNEL) staining of tumor sections. As shown in Fig. 4g, Cu@cLAs-mediated therapy induced severer apoptosis and necrosis of the tumor relative to those of DOX-treated mice. Consistent with the therapy results, according to the survival experiment, 80% of mice injected with Cu@cLAs survived at 40 days, whereas mice injected with saline and DOX all died at 24 and 36 days (Fig. 4e).

The Cu@cLAs group showed good biosafety during the treatment, as indicated by the absence of significant weight changes (Fig. 4f). Interestingly, it was found that several obvious myocardial injuries were observed in the animals treated with DOX, as revealed by the images of the corresponding cardiac H&E staining (Supplementary Fig. S13). Overall, these results indicated the high anticancer potency and excellent biosafety of Cu@cLAs.

## Conclusions

In conclusion, a copper-mediated CDT agent with extremely low Cu usage has been developed by anchoring copper on cross-linked LA nanoparticles. The cyclic regeneration was confirmed by rapid and efficient *in situ* generation of continuous ROS via the recycling between the redox couples of LA/DHLA and Cu^2+^/Cu^+^. The recycling was confirmed by measuring intracellular Cu and DHLA content. They kept at high levels over a long time in Cu@cLAs group while the Cu and DHLA in free cLAs and Cu^2+^ groups were largely metabolized. Besides, the persistent generation of intracellular ROS level was also verified. The *in vivo* antitumor experiment based on the MCF-7/R nude mice model showed that the TIR of the Cu@cLAs was up to 77.9%, much better than that of the first-line chemotherapy drug DOX**·**HCl (TIR: 42.9%). Moreover, the copper content of 5 mg kg^−1^ Cu@cLAs was only 0.05 mg kg^−1^, 32-fold lower than that of the most efficient copper-containing CDT nanosystem (1.6 mg kg^−1^) reported so far. Benefited from its efficient anticancer activity, the further survival experiment suggested that 80% of mice injected with Cu@cLAs survived at 40 days, whereas mice injected with saline and DOX all died at 24 and 36 days. Featured by the extremely low Cu usage, good biosafety and simple components and efficient anticancer activity, Cu@cLAs holds good potential for clinical transformation. Additionally, although the self-cyclic strategy is only used for solve the problem of excessive copper usage in tumor CDT, this concept is generally applicable and may extend to other disease therapies with the high metal consumption.

## Supplementary Material

rbad021_Supplementary_Data
