# Supplementary material for: Copper-mediated chemodynamic therapy with ultra-low copper consumption by doping cupric ion on cross-linked (R)-(+)-lipoic acid nanoparticles
Source: Regen Biomater. 2023 Mar 23;10:rbad021. doi: 10.1093/rb/rbad021 (PMC10070036; doi:10.1093/rb/rbad021)
Supplement: rbad021_Supplementary_Data [file rbad021_supplementary_data.docx]

***<Supporting information>***

Copper-Mediated Chemodynamic Therapy with Ultra Low Copper Consumption by Doping Cupric Ion on Cross-Linked (*R*)-(+)-Lipoic Acid Nanoparticles

Rong Cui,^‡a^ Bing Li,^‡b^ Chunyan Liao,^a^ and Shiyong Zhang,*^a^

^a^ College of Biomedical Engineering and National Engineering Research Center for Biomaterials, Sichuan University, 29 Wangjiang Road, Chengdu 610064, China
^b^Hubei  Key Laboratory of Wudang Local Chinese Medicine Research, School of  Pharmaceutical Sciences, Hubei University of Medicine, Shiyan, Hubei 442000, China

^‡^ Rong Cui and Bing Li contributed equally to this work.

^*^To whom correspondence should be addressed. S. Zhang, E-mail: [szhang@scu.edu.cn](mailto:szhang@scu.edu.cn); Phone: +86-28-85411109. Fax: +86-28-85411109.

***General Method***

The particle sizes were measured with a dynamic light scattering (DLS) analyzer (Malvern Zetasizer Nano ZS90). SEM studies were performed on a Hitachi SU8100 instrument, operating at 3.0 kV. EDS spectrums were determined by JSM-5900LV (Japan) instrument. The content of copper element was obtained by ICP-AES (IRIS Advantage). The valence state of the copper element was obtained by X-ray photoelectron spectroscopy (XPS, XSAM800). The UV-vis was measured using a Shimadzu UV-2600 instrument. The cytotoxicity was evaluated with a tetrazolium-based colorimetric assay (MTT test). The cell viability was measured using a Thermo Varioskan Flash Microplate Reader (USA). Cell imaging was performed under a confocal laser scanning microscope (CLSM, Leica TCP SP5) or an inverted microscope. High performance liquid chromatography-mass spectrometry (HPLC-MS) analysis was performed with a Shimadzu LCMS-2020 System (Shimadzu, Kyoto, Japan).

*Materials:* The reagents used in this word, unless otherwise noted, were provided by commercial suppliers. Deionized water was used in all aqueous experiments. Copper (II) chloride dihydrate and acetone were purchased by chengdu Kelong Co. Ltd. Lipoic acid was purchased from Adamas-beta Co. Ltd. Hydrogen peroxide, methylene blue, and L-glutathione were bought from Aladdin (Shanghai, China). Acetoxymethyl (AM), propidium iodide (PI), and thiazolyl blue (MTT) were bought from Aladdin (Shanghai, China). The adriamycin (ADR)-resistant human breast cancer cell line MCF-7 (MCF-7/R) were purchased from BeNa Culture Collection (Beijing, China) and was cultured in the Roswell Park Memorial Institute (RPMI-1640) medium with the addition of ADR (0.1 ug mL^−1^). The FBS was purchased from Biological Industries (Israel). The reactive oxygen species (ROS) assay kit, and the bicinchoninic acid (BCA) protein assay kit were purchased from Beyotime (Shanghai, China). The BALB/c nude animals were obtained through the Institute of Laboratory Animals of Sichuan Academy of Medical Sciences & Sichuan Provincial People’s Hospital, and the corresponding animal experiments were performed according to the “Principles of Laboratory Animal Care” (NIH) guidelines and approved by the Animal Care Committee of Sichuan University (Chengdu, China).

***Synthesis***

**Synthesis of cross-linked lipoic acid nanoparticles (cLAs).** A solution of (*R*)-(+)-lipoic acid (50 mg, 0.24 mmol) in acetone (5 mL) was added dropwise into 50 mL of deionized water at room temperature under shaking. After that, the acetone was removed by a rotary evaporator. The resulting solution was then irradiated under ultraviolet light at 365 nm for 4 h and stirred at room temperature by a magnetic stirrer overnight. Finally, the reaction mixture was dialyzed with deionized water at 37 °C for 48 h to obtain the cLAs (Spectra/Pore, MWCO 1000) as a pale blue emulsion.

**Copper content determination of Cu@cLAs.** The copper content of the resulting Cu@cLAs was determined by ICP-AES assay. Briefly, Cu@cLAs (10 mg) were dissolved in conc. HNO3 and shaken for 24 h. The resulting supernatant was collected and Cu analysis was carried out by ICP-AES with calibration against Cu standard solutions.

***In vitro* dilution stability assay.** The dilution stability of Cu@cLAs was assessed through diluting the LA below its CAC concentration (17.3 µg/mL). Briefly, the concentration of LA in Cu@cLAs was diluted to 784, 392, 196, 49, 24.5 and 13.3µg/mL, sequentially. Then, the sizes of the nanoparticles were detected by DLS.

***In vitro* fetal bovine serum (FBS) stability assay.** The FBS stability of Cu@cLAs was studied through incubating with FBS (10%, v/v). Briefly, 9 mL of Cu@cLAs was incubated at 37 °C with 1 mL of FBS. Then, the sizes of the nanoparticles at 0 h, 2 h, 4 h, 6 h, 8 h, 10 h and 12 h were detected by DLS.

***In vitro* degradation assay****.**The Cu@cLAs release was assumed to start as soon as the dialysis bags were placed into the reservoir. Briefly, dialysis bags (Spectra/Pore, MWCO 1000) containing 2.0 mL of Cu@cLAs were immersed in 48 mL PBS (pH = 7.4) with 10 mM GSH, respectively. At predetermined periods (0 h, 2 h, 4 h, 6 h, 8 h, 10 h and 12 h), 0.5 mL of the solution was obtained periodically from the reservoir, and the amounts of released Cu@cLAs were analyzed by DLS with Count rate.

***In vitro* cytotoxicity assessment.** To assess the *in vitro* anticancer activities of cLAs, Cu^2+^ and Cu@cLAs with various Cu/LA ratios, MCF-7/R cells were seeded in 96-well culture plates with a density of 5×10^3^ cells per well, and incubated at 37 °C, 5% CO2. After 24 h incubation, the culture media was removed, and the fresh media (100 µL) containing various concentrations of cLAs, Cu^2+^ and Cu@cLAs with various Cu/LA ratios were added to each well, respectively. After 48 h incubation, the old culture media were removed and 100 µL fresh media containing 20 µL of MTT solution (5 mg mL^-1^) was added to each well. The cells were allowed to incubate for another 4 h, then carefully removed the culture media and added 150μL dimethyl sulfoxide to each well to fully dissolve the formed blue formazan crystals. The absorbance at 490 nm of each well was determined by a microplate reader, Varioscan Flash. The MTT assay was also utilized to estimate the anticancer activities of Cu@cLAs against MCF-7/R cells.

***In vitro* observations of living and dead cells after different treatments.** MCF-7R cells seeding in 6-well plates at a density of 8000 cells per well were cultured for 24 h at 37 °C. Cu^2+^ (1 µM), cLAs ([LA] = 100 μM), Cu@cLAs (Cu: 1µM, LA: 100 μM) were then sequentially added to each well. After 4 h of coincubation, the cells were costained by 20 mM calcein AM and 20 mM PI and imaged under an inverted fluorescence microscope.

**Western blot assay.** The expression levels of bcl-2 protein and β-actin in MCF-7R cells after treatment with Cu^2+^ (1 µM), cLAs ([LA] = 100 μM), Cu@cLAs (Cu: 1µM, LA: 100 μM) for 3 h were detected by the western blot assay. The detailed experimental procedures have been discussed in our previous report.

**Detection of ROS using methylene blue (MB).** The buffer solution containing MB (10 μM) were mixed with Cu@cLAs (0.5 mM) plus GSH (10 mM) and H_2_O_2_ (100µM). The mixing system was adjusted to a pH of 7.4 and incubated for 2h at 37 °C, and then the absorbance of MB was measured by UV-vis spectroscopy. The MB solutions treated with Cu@cLAs (1 mg/mL) or H_2_O_2_ (100µM) alone or Cu@cLAs (1 mg/mL) plus GSH (10 mM) were used as control groups.Solutions of 10 μM MB, 10 mM H_2_O_2_, and Cu@cLAs (1 mg/mL) plus GSH (10 mM) were formed at different pH values. After different times, ROS-induced MB degradation was measured by the change in absorbance.

**Detection of •OH generation.** Briefly, 50 μL of 5,5-dimethyl-1-pyrroline-N-oxide (DMPO) solution (100 mM) was added into the reaction groups containing the following additions: Cu@cLAs (1 mg/mL ) + GSH (10 mM) + H_2_O_2_ (100 µM) at pH 7.4; Cu@cLAs (1 mg/mL) + GSH (10 mM) at pH 7.4 and H_2_O_2_ (100 µM) at pH 7.4. After incubating at 37 °C for 3 h, 2 μL of the above samples was used for the electron spin resonance (ESR) analysis.

**Intracellular ROS assessments.** Intracellular ROS levels were determined by 2,7-dichlorodihydrofluorescein diacetate (DCFH-DA) staining. To obtain confocal images of ROS-induced DCF fluorescence, MCF-7R cells seeding in glass-bottom petri dishes at a density of 8000 cells per plate were cultured for 24 h at 37 °C. MCF-7R cells were incubated with Cu^2+^ (1 µM), cLAs ([LA] = 100 μM), Cu@cLAs (Cu: 1µM, LA: 100 μM) and stained with DCFH-DA for 30 min in a medium without serum according to the recommended procedure of the reactive oxygen species assay kit. After staining the cell nuclei for 15 min with Hoechst 33342 and washing with PBS three times, the cell images were obtained on CLSM. The fluorescence of DCF was collected at 523 nm with excited wavelength of 488 nm.

**Intracellular H_2_O_2_ assessments.** MCF-7/R cells (1.0 × 10^5^/well) were seeded into the 60 mm culture plate and cultured for 24 h at 37 ℃/5% CO_2_. Then, cells were incubated with medium or Cu@cLAs for different times (0, 2, 4, 6, 8, 10, 12 h), followed by removal of medium or materials and cleaning with PBS for 3 times. At last, the cells were collected and the intracellular H_2_O_2_ was detected by using the hydrogen peroxide assay kit.

**Hemolytic test.** The hemolytic experiment was performed to assess the hemocompatibility of Cu@cLAs. Briefly, 950 µL of Cu@cLAs nanoparticle solution at the preset concentrations (1, 2, 5, 7, and 10 mg mL^-1^) were added into 50 µL red blood cells (RBC) suspension (10%, v/v) and incubated in a shaking water bath at 37 °C. Distilled water and saline were set as positive and negative controls, respectively. After 2 h incubation, samples were collected and centrifuged for 10 min at 1500 rpm. The supernatant solution which showed red color meant hemolytic positive. On the contrary, absolute achromatic of the supernatant meant hemolytic negative. The absorbance of the supernatant solution was determined by UV-vis spectra at 542 nm.

**Hemagglutination assay.** The hemagglutination assay was also adopted to assess the hemocompatibility of Cu@cLAs. Briefly, 950 µL of Cu@cLAs nanoparticle solution at the preset concentrations (1, 2, 5, 7, and 10 mg mL^-1^) were added into 50 µL RBC suspension (2%, v/v) in 24-well plates, respectively. The 2% RBC suspension that treated with saline was set as negative control. After 2 h incubation at room temperature, images were taken under a microscope at a magnification of 400×.

***In vivo* acute toxicity test.**Acute toxicity was used to obtain the median lethal dose (LD_50_) of the Cu@cLAs. Fifty Balb/c mice were divided into 5 groups (5 males and 5 females in each group) and maintained under the standard conditions with free access to food and water. After one-week acclimation, the solutions of Cu@cLAs in sterile PBS (0.2 mL) were injected at the preset doses via the tail vein. Animals were observed every 2 h on the treatment day, twice a day later, for up to 15 days post injection. The death of mice was recorded and the LD_50_ and 95% confidence interval were calculated by the Bliss method.

***In vivo* anticancer efficacy evaluation.** Five week-old female balb/c nude mice were implanted subcutaneously MCF-7/R cells (2 × 10^6^ cells/site). When the tumor volume reached about 50 mm^3^, an *in vivo* cascade anti-tumor experiment was conducted. All mice were randomly divided into three groups. The mice bearing tumors were randomly divided into three groups (n = 5) and intravenously injected with 100 μL saline, Dox (5mg/kg), Cu@cLAs (5 mg/kg). The tumor volumes and body weights in each group were measured and recorded every 3 days. After 21 days of the corresponding treatments, all nude mice were dissected and histopathologically analyzed.

**Histological and Immunohistochemical Analyses.** After 21 days of treatment, tumors and major organs of mice were collected and fixed with paraformaldehyde for 48 h. The histopathological and immunohistochemistry analyses were performed after hematoxylin and eosin (H&E) and terminal 2′-deoxyuridine 5′-triphosphate nickend labeling (TUNEL) staining.


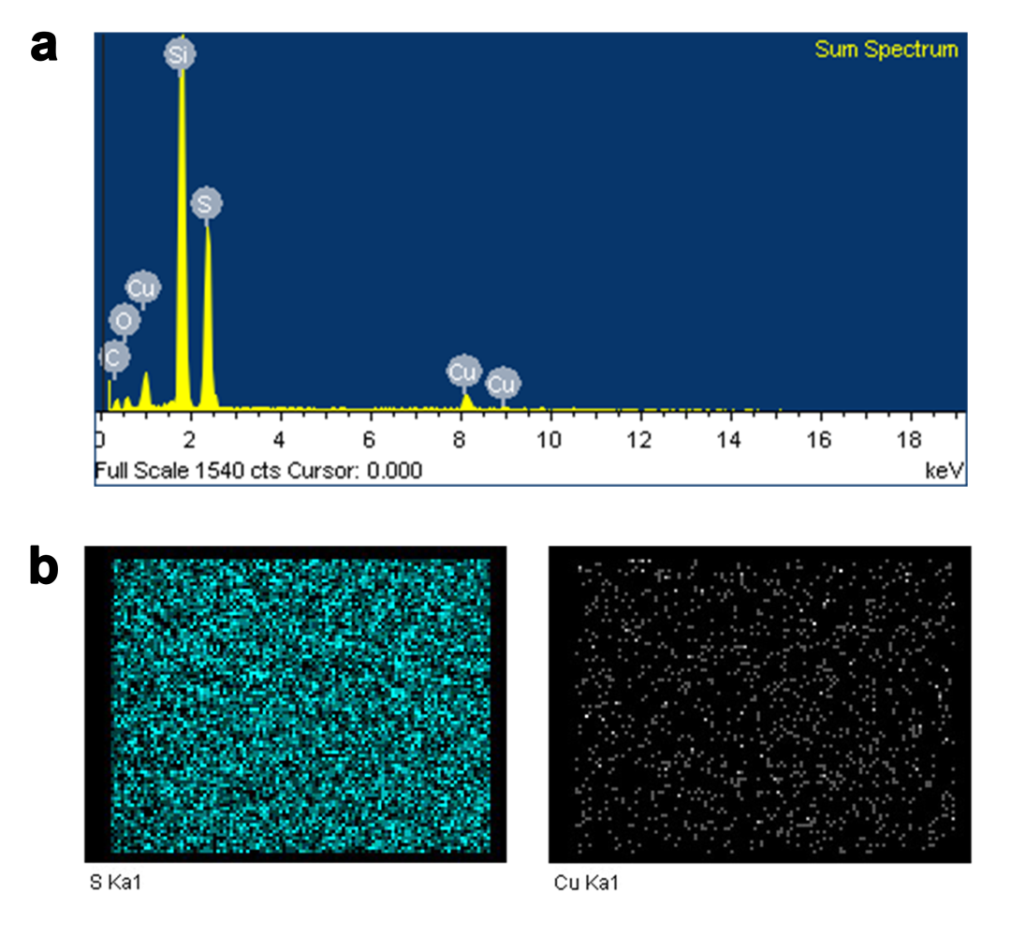


**Figure S**1. EDS analysis of Cu@cLAs. (a) EDS Spectra and (b) S, Cu elemental mapping.


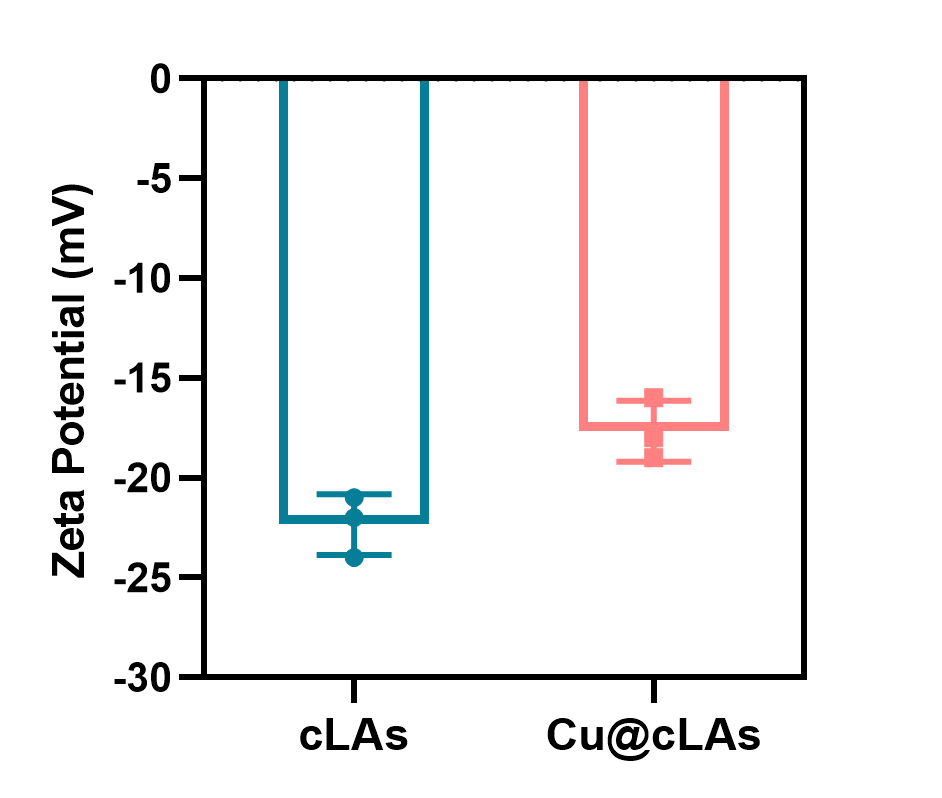


**Figure S**2. Zeta potentials of cLAs and Cu@cLAs determined by dynamic light scattering.


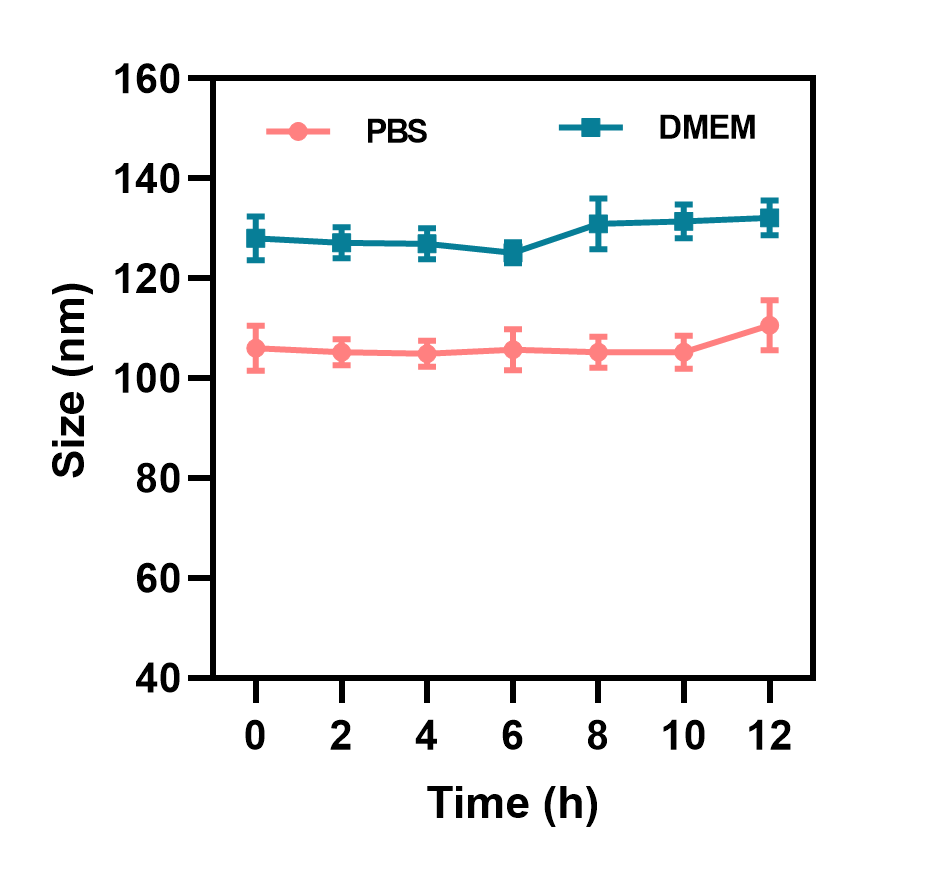


**Figure S**3. Particle size of Cu@cLAs incubated with PBS and DMEM as a function of time, respectively.


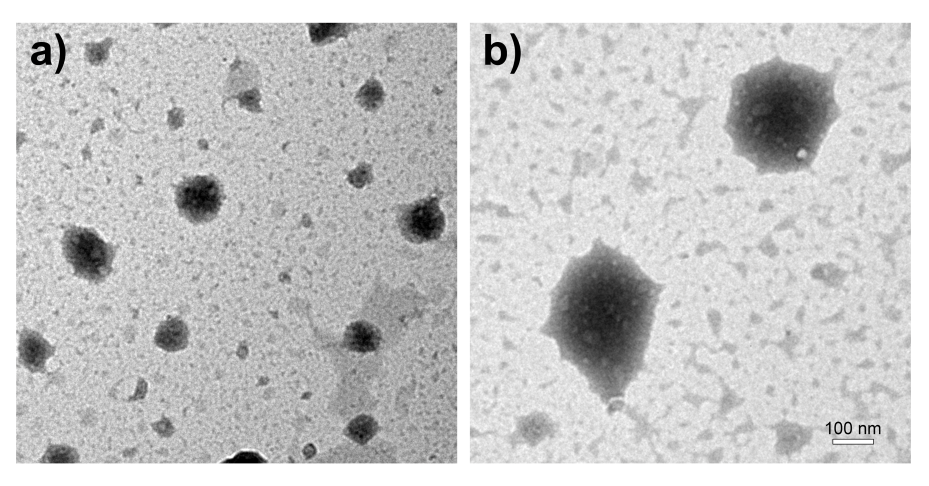


**Figure S**4. The TEM images of (a) Cu@cLAs and (b) Cu@cLAs incubated with GSH for 2 h.


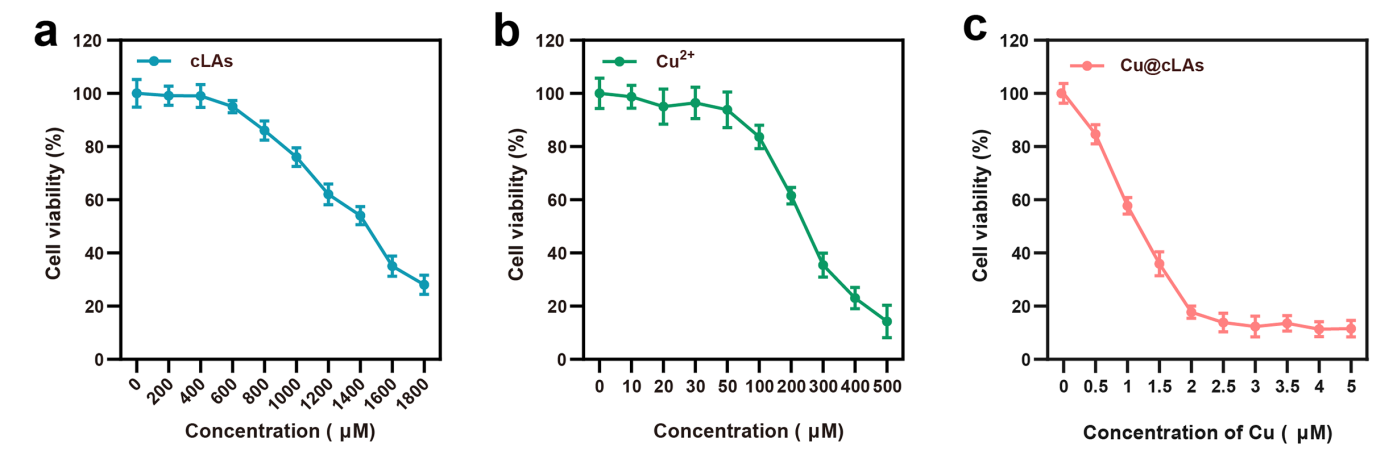


**Figure S5.** Cell viability of MCF-7/R cells after incubated with cLAs (a), Cu^2+^ (b) and Cu@cLAs (c) after incubation at various concentrations at 37°C for 48 h (mean ± SD, n = 5).


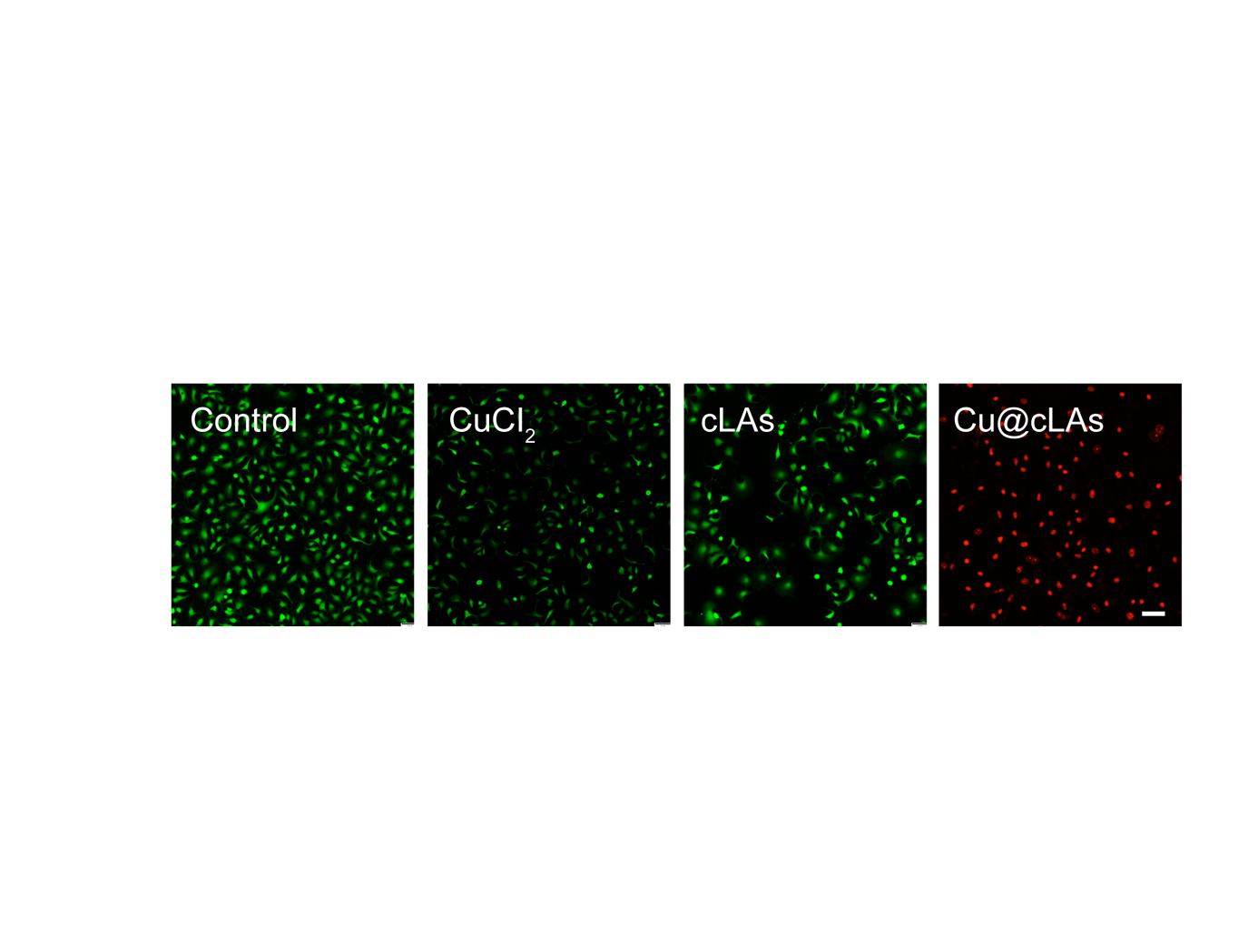


**Figure S**6. Live/dead staining of MCF-7R cells treated with various concentrations of Cu@cLAs for 48 h at 37 °C, 5% CO_2_. Scale bar : 100 µm.


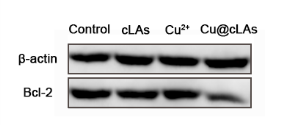


**Figure S**7. Western blot analysis for protein levels of Bcl-2 expression in MCF-7R cells after incubation with cLAs , CuCl_2_ and Cu@cLAs for 3 h. β-actin was used as loading control.


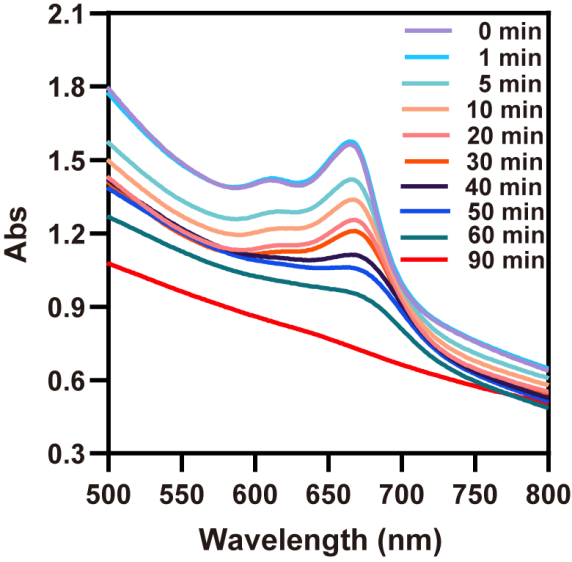


**Figure S**8. The degradation process of MB at different time points at pH=7.4.


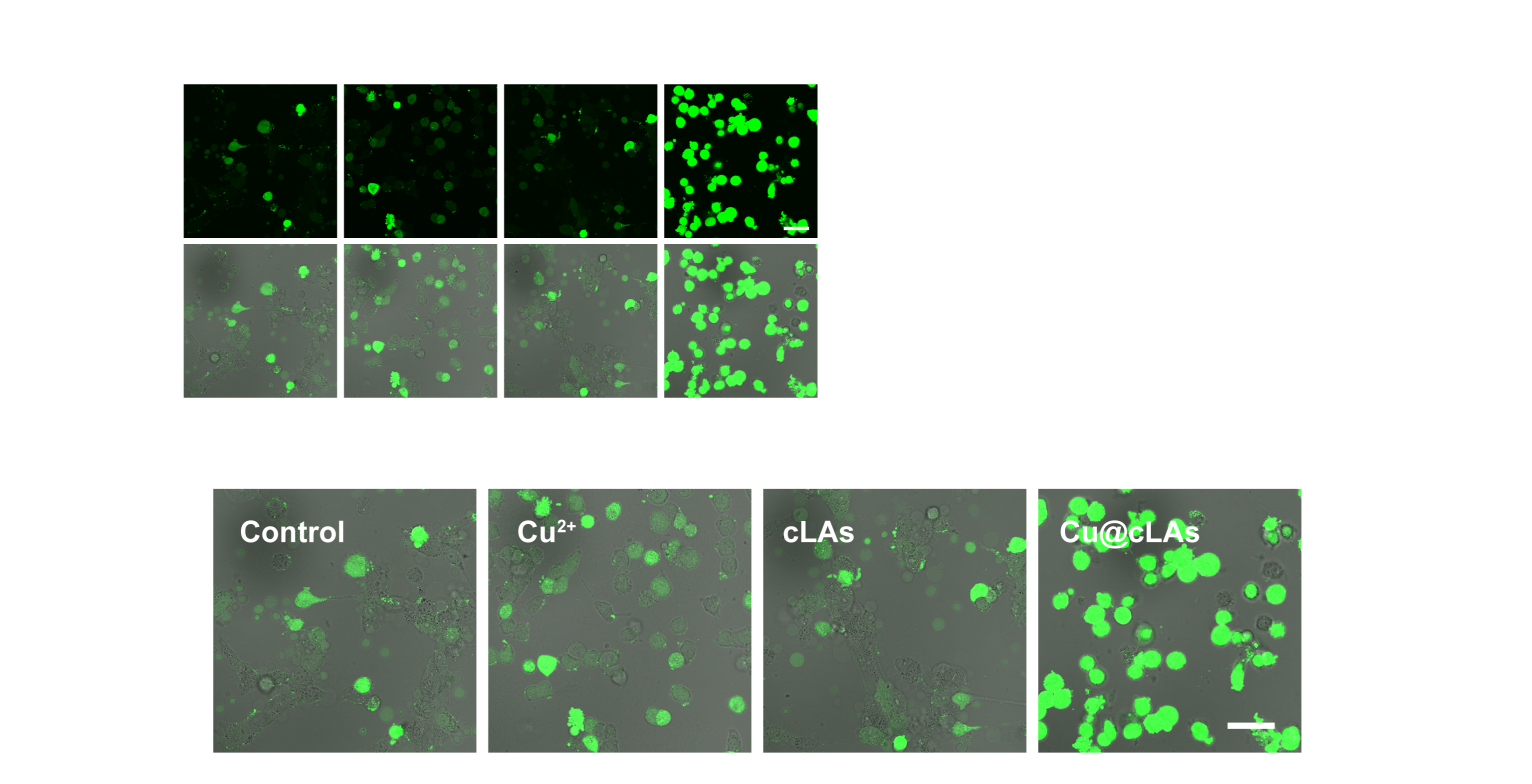


**Figure S**9. CLSM images of ROS production in MCF-7R cells incubated with the indicated materials for 3 h. Scale bar: 25 µm.


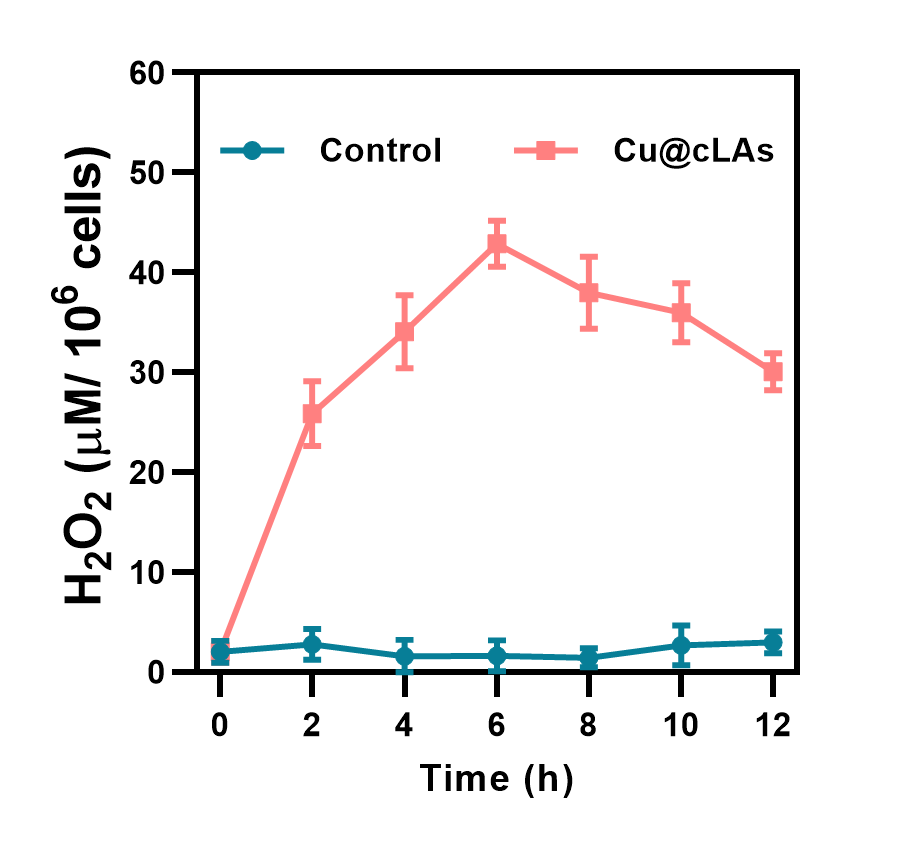


**Figure S**10. The content of intracellular H_2_O_2_ in MCF-7R cells incubated with Cu@cLAs for different times.


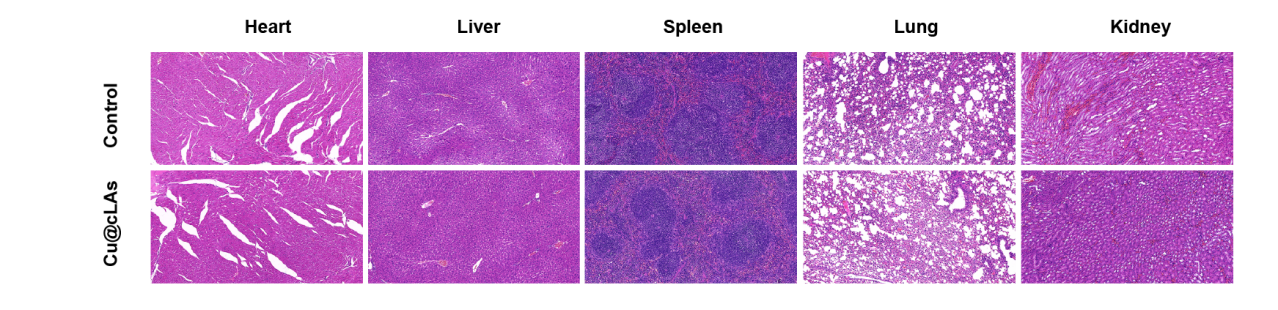


**Figure S**11. Hematoxylin and eosin (H&E) staining assay of heart, kidney, liver, lung, and spleen tissues of mice after i.v. administration of 50 mg kg^-1^ Cu@cLAs or saline (n = 5).


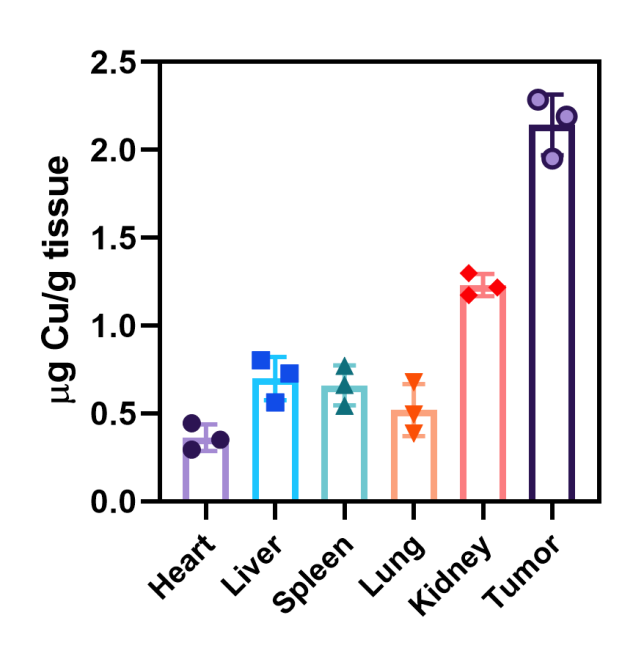


**Figure S**12. Biodistribution of Cu in MCF-7R tumor-bearing nude mice after injection with Cu@cLAs (mean ± SD, n = 3).


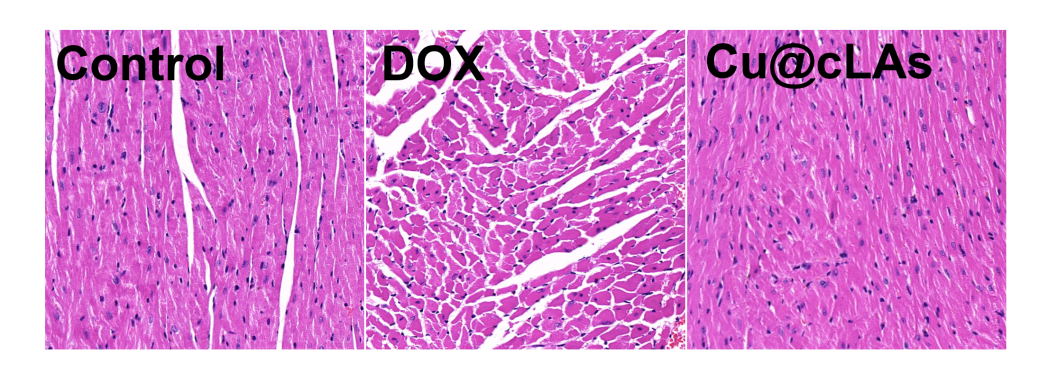


**Figure S**13. Histological analyses of the heart tissue after therapy.

**Table S1.** The size and PDI of cLAs and Cu@cLAs

| cLAs | | Cu@cLAs | |
| --- | --- | --- | --- |
| Size (nm) | PDI | Size (nm) | PDI |
| 78.73 | 0.262 | 104.38 | 0.205 |
| 79.29 | 0.398 | 108.29 | 0.238 |
| 75.78 | 0. 258 | 106.78 | 0.332 |

PDI: particle distribution index.

**Table S2.** IC_50_ of Cu^2+^, cLAs, and Cu@cLAs against B16 and 4T1 cells at 37 °C for 48 h.

| Drug formulation | ICCu 50 [10^-6^ M) | | | ICcLAs 50 [10^-6^ M] ^a^ | |
| --- | --- | --- | --- | --- | --- |
|  | 4T1 | B16 | 4T1 | | B16 |
| Cu^2+^ | 276.98 | 328.34 | ‒ | | ‒ |
| cLAs | ‒ | ‒ | 1978.31 | | 2362.63 |
| Cu@cLAs | 7.64 | 11.85 | 764 | | 1185 |

^a^ The IC_50_ of cLAs was calculated by the molecular mass of LA.

**Table S3.** Serum biochemistry analysis for mice with i.v. administration of Cu@cLAs at the concentration of 50 mg kg^-1^ (mean ± SD, n = 5). Saline treated mice were used as control group.

| Indicator | Saline | Cu@cLAs | Normal range |
| --- | --- | --- | --- |
| ALT (U/L) | 39.46 ± 5.90 | 50.43 ± 5.27 | 30-100 |
| AST (U/L) | 95.71 ± 7.65 | 113.81 ± 6.54 | 60-220 |
| CRE (μmol/L) | 52.95 ± 3.98 | 84.24 ± 6.47 | 22-97 |
| BUN (mmol/L) | 25.52± 2.58 | 23.52 ± 1.61 | 18-33 |
